# Integrating Expression Data-Based Deep Neural Network Models with Biological Networks to Identify Regulatory Modules for Lung Adenocarcinoma

**DOI:** 10.3390/biology11091291

**Published:** 2022-08-30

**Authors:** Lei Fu, Kai Luo, Junjie Lv, Xinyan Wang, Shimei Qin, Zihan Zhang, Shibin Sun, Xu Wang, Bei Yun, Yuehan He, Weiming He, Wan Li, Lina Chen

**Affiliations:** 1 College of Bioinformatics Science and Technology, Harbin Medical University, Harbin 150000, China; 2 Department of Respiratory, Second Affiliated Hospital of Harbin Medical University, Harbin 150000, China; 3 Institute of Opto-Electronics, Harbin Institute of Technology, Harbin 150000, China

**Keywords:** regulatory module, deep neural network, competing endogenous RNA, lung adenocarcinoma, biological network

## Abstract

**Simple Summary:**

The growing evidence suggested that competing endogenous RNAs (ceRNAs) have significant associations with tumor occurrence and progression, yet the regulatory mechanism of them in lung adenocarcinoma remains unclear. Identification of the regulatory modules for lung adenocarcinoma is a critical and fundamental step towards understanding the regulatory mechanisms during carcinogenesis. Deep neural network (DNN) models have become a powerful tool to intelligently recognize the sophisticated relationships of ceRNAs appropriately. In this paper, multiple deep neural network models were constructed using the expression data to identify regulatory modules for lung adenocarcinoma in biological networks. Three identified regulatory modules association with lung adenocarcinoma were validated from three aspects, i.e., literature review, functional enrichment analysis, and an independent dataset. The regulatory relationships between RNAs were validated in various datasets, including CPTAC, TCGA and an expression profile from the GEO database. Our study will contribute to improving the understanding of regulatory mechanisms in the carcinogenesis of lung adenocarcinoma and provide schemes for identifying novel regulatory modules of other cancers.

**Abstract:**

Lung adenocarcinoma is the most common type of primary lung cancer, but the regulatory mechanisms during carcinogenesis remain unclear. The identification of regulatory modules for lung adenocarcinoma has become one of the hotspots of bioinformatics. In this paper, multiple deep neural network (DNN) models were constructed using the expression data to identify regulatory modules for lung adenocarcinoma in biological networks. First, the mRNAs, lncRNAs and miRNAs with significant differences in the expression levels between tumor and non-tumor tissues were obtained. MRNA DNN models were established and optimized to mine candidate mRNAs that significantly contributed to the DNN models and were in the center of an interaction network. Another DNN model was then constructed and potential ceRNAs were screened out based on the contribution of each RNA to the model. Finally, three modules comprised of miRNAs and their regulated mRNAs and lncRNAs with the same regulation direction were identified as regulatory modules that regulated the initiation of lung adenocarcinoma through ceRNAs relationships. They were validated by literature and functional enrichment analysis. The effectiveness of these regulatory modules was evaluated in an independent lung adenocarcinoma dataset. Regulatory modules for lung adenocarcinoma identified in this study provided a reference for regulatory mechanisms during carcinogenesis.

## 1. Introduction

Lung cancer is one of the most common causes of cancer deaths in the world [1], of which the most common type is lung adenocarcinoma that comprises about 40% of all lung cancer cases. It remains one of the most aggressive and lethal tumor types [2], despite the understanding of the pathogenesis and new treatments for lung adenocarcinoma having improved [3]. Many experiments have confirmed that regulations of RNA molecules are closely related to the occurrence and development of lung adenocarcinoma [4]. Therefore, regulatory modules of lung adenocarcinoma identified based on biological networks and expression data are beneficial to understand the carcinogenesis.

Most proteins activate and function through their interactions. Thus, protein interactions and their networks are very important in most biological functions and processes [5]. Protein interaction networks can help us better understand the disease process, which is of great significance in the identification of disease proteins/gene [6,7]. Roudi et al. identified differentially expressed genes (DEGs) at each stage of lung adenocarcinoma in four datasets from the Gene Expression Omnibus (GEO) database. Co-expression clusters and biological pathways were identified for common and unique DEGs, respectively. Five hub genes crucial for lung adenocarcinoma were observed from a protein interaction network of common DEGs among all stages, and confirmed using an independent dataset collected from The Cancer Genome Atlas (TCGA) [8]. Better understanding of diseases could be obtained through disease proteins/genes identified from protein interaction networks.

A competing endogenous RNA (ceRNA) network links the function of protein-coding mRNAs with the function of non-coding RNAs (ncRNAs, including microRNAs (miRNAs) and long non-coding RNAs (lncRNAs)) [9], which better explains the respective roles of different RNAs in biological processes. Dysregulation of their expression has been implicated in various diseases, including cancer. For example, Jafarinejad-Farsangi et al. predicted top miRNAs targeting SARS-CoV-2 genome and differentially expressed genes (DEGs) in the lungs of patients infected with SARS-CoV-2 [10]. Hsa-mir-130a has been proven by many clinical trials and bioinformatics studies to be a marker gene that widely participated in various types of tumors by down-regulating a variety of key proto-oncogenes [11]. Li et al. identified 11 gastric cancer-specific lncRNAs, 9 miRNAs, and 41 mRNAs from a gastric cancer ceRNA network generated from miRcode and miRTarBase based on bioinformatics [12]. These ncRNAs have been extracted through ceRNA regulatory relationships and served as determined or potential tumor suppressors or therapeutic targets [13]. A better understanding of the underlying mechanisms of these regulations and their roles in cancer initiation is essential for the development of more robust clinical diagnostic tools.

In the field of biology, deep learning has greatly improved the reliability of biological big data analysis, when compared to traditional machine learning with the advantages of self-learning and high generalization ability. The evaluation criteria obtained by deep learning using a large volume of patient data not only have a wide range of confidence, but also greatly reduce the cost of clinical diagnosis [14]. Deep neural network (DNN) models have become a powerful tool of deep learning and artificial intelligence. Good progress has been made in the application of DNNs in various biological branches, and the diagnosis applying DNNs can even reach the level of experienced clinical experts [15,16,17].

**Figure 1:**
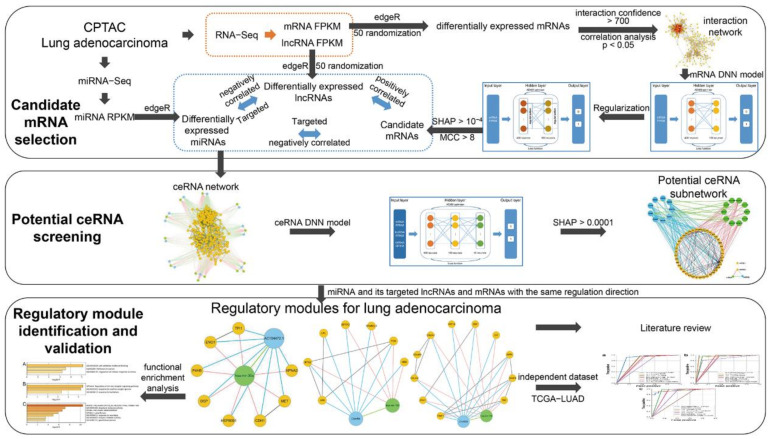
The workflow of this study. Firstly, expression data of mRNAs, miRNAs and lncRNAs were extracted from CPTAC, and differential analysis was performed to obtain significant differentially expressed mRNAs, miRNAs and lncRNAs. Protein interaction and co-expression analysis were performed on differential mRNAs to construct the interaction network, and DNN models were established to screen out candidate mRNAs with significant contribution to the DNN models. In addition, multiple experimentally validated miRNA target databases were combined for these candidate mRNAs, differential miRNAs and lncRNAs to form a ceRNA network. Then a ceRNA DNN model was performed to identify potential ceRNAs. Finally, modules comprised of miRNAs and their regulated mRNAs and lncRNAs with the same regulation direction were identified as regulatory modules for lung adenocarcinoma from the potential ceRNA subnetwork. These valuable tumor regulatory modules were validated by literature review, functional enrichment analysis and an independent lung adenocarcinoma dataset from The Cancer Genome Atlas (TCGA-LUAD).

In this paper, regulatory modules for lung adenocarcinoma were identified using multiple DNN models in interaction and ceRNA networks (Figure 1). This would not only improve the ability in the identification of regulatory modules for lung adenocarcinoma, but also provide a basis for the understanding of regulatory mechanisms during carcinogenesis.

## 2. Materials and Methods

### 2.1. Data

In this study, RNA-Seq data of 204 samples (including 102 tumor tissues and matched paracancerous non-tumor tissues) and miRNA-Seq data of 202 samples (including 101 tumor tissues and matched paracancerous non-tumor tissues) for lung adenocarcinoma were acquired from the Clinical Proteomic Tumor Analysis Consortium (CPTAC) project. A total of 100 pairs of tumor tissues and matched paracancerous non-tumor tissues) shared by RNA-Seq and miRNA-Seq data were selected for data analysis. The pathological characteristics of the lung adenocarcinoma patients are presented in Table 1.

For RNA-Seq data, the Ensemble database was used to annotate symbol and gene_biotype attributes. According to the gene_biotype attribute, 14,560 lncRNAs and 19,450 mRNAs were extracted. Fragments per kilobase of exon per million reads mapped (FPKM) values were chosen as the representative measure of mRNA or lncRNA expression. Read counts for 1886 miRNAs were first obtained from the miRNA-Seq data. Then Reads Per Kilobase per Million mapped reads (RPKM) values were calculated for each sample using the Reads per million mapped reads (RPM) for each miRNA.

The data have undergone quality control and normalization using the edgeR TMM method after removing RNAs with more than 1/3 of the missing values for RNA-Seq data or 2 missing values for miRNA-Seq data. Then edgeR was used to detect significant differentially expressed mRNAs, lncRNAs and miRNAs between lung adenocarcinoma and corresponding paracancerous non-tumor tissues based on their read counts. |log2(fold-change)| >1 and FDR adjusted *p* value < 0.05 were used as thresholds.

In order to ensure the stability and reliability of the obtained significant differentially expressed mRNAs and lncRNAs, due to their large size, 4/5 of the samples were randomly selected each time to detect significant differentially expressed mRNAs and lncRNAs. The intersection of the differentially expressed mRNAs and lncRNAs obtained after 50 times of randomization was taken as the significant differentially expressed mRNAs and lncRNAs. Because of the small number of miRNAs and sparse expression values, the differential expression analysis was performed one time to obtain significant differentially expressed miRNAs [18]. A total of 4888 significant differentially expressed RNAs, including 3399 mRNAs (1463 up-regulated and 1936 down-regulated), 1098 lncRNAs (519 up-regulated and 579 down-regulated) and 391 miRNAs (330 up-regulated and 61 down-regulated) were obtained.

### 2.2. DNN Model

#### 2.2.1. DNN Model Feature

The interaction network for significant differentially expressed mRNAs was constructed according to interactions between proteins they encoded and their expression correlation. Interactions with confidence >700 from the STRING database and significantly co-expressed (*p*-value < 0.05) were screened out. An interaction network containing 1122 mRNAs and 13455 pairs of interactions was obtained. The FPKM values of these 1122 mRNAs were the features for the mRNA DNN models.

Based on experimentally validated miRNA targets (mRNAs and lncRNAs) from TarBase, mirTarBase, miRrecords, targetScan and starBase, the ceRNA triplets (mRNA-miRNA-lncRNA) were constructed if miRNAs were negatively correlated with lncRNAs and mRNAs, respectively, and mRNAs positively correlated with lncRNAs (Z-Score > 0, *p*-value < 0.05). The ceRNA network was constructed accordingly. The FPKM/RPKM values of these mRNAs, miRNAs and lncRNAs were employed as the features for the ceRNA DNN models.

#### 2.2.2. DNN Model Construction

Multiple fully connected DNN models were built with Google TensorFlow 2.0 architecture. The architecture of each fully connected DNN model is composed of an input layer, multiple hidden layers, and an output layer. The output layer with 1/0 label indicates the sample to be a cancer one or not. The Adaptive Moment Estimation (ADAM) optimizer with default parameters supplied by Keras was chosen due to the small sample size [19]. Here, the loss function of binary cross-entropy was used.

The performance of a DNN model is associated with many model training related parameters, including batch size, number of epochs and the learning rate in the training process. The model training requires the accumulation of multiple learning rounds. In each round, a batch of training sets will be randomly selected according to the learning rate. The more samples each batch has, the faster the convergence of the model will be, and the weaker the generalization ability will be. Since the purpose of this analysis was to obtain regulatory modules for lung adenocarcinoma, and the sample size and characteristic number were relatively small, the batch size = 16 and epoch >= 1000 was set. The learning rate should be high to prevent under-fitting if the batch number is large and be low to prevent overfitting when the batch number is small. Therefore, the learning rate was set to 0.0001 for more learning epochs.

In order to prevent overfitting in the learning process, DNNs could be further optimized from two aspects: regularization layer and Drop layer. In the regularization layer, each multidimensional feature is regularized after the neuron layer to make the gap smaller and reduce the dependence of high-weight features; while in the Drop layer, some eigenvalues are discarded randomly. In order to preserve the integrity of features, regularization was used to prevent overfitting in this paper.

Features that contributed significantly to the DNN models were more biologically significant. SHapley Additive exPlanations (SHAP) is a game-theoretic approach to explain the output of a machine learning model. The SHAP value represents the contribution of a feature to the machine learning model. In this paper, DeepExplainer of the python SHAP module was used to approximate SHAP values for the DNN models [20]. The impact of each feature on each sample was obtained using force_plot. Then the arithmetic average of absolute values for the impact representing the importance of the feature to all samples, denoted as the SHAP value, was calculated by summary_plot. The larger the SHAP value is, the greater the contribution of the feature to the corresponding DNN model. The identification of candidate and potential RNAs in this paper was based on the SHAP values of RNAs in DNN models.

#### 2.2.3. DNN Model Evaluation

The samples were split randomly into a 70% training set and 30% validation set. The randomization was repeated 50 times. The model was then evaluated with the closeness between the expected output and the actual output by two measures: accuracy and loss function. Accuracy indicates the precision of deep learning. The closer the accuracy approaches to 1, the more accurate the prediction will be. The accuracy of training sets is generally higher than that of validation sets. The loss represents the degree of deviation between the training or validation results and the real results. The closer to 0, the better the fitting with the real results will be. The loss of the validation sets is generally not less than that of the training sets. DNN models were evaluated via the accuracy and loss curves for the training and validation sets.

### 2.3. Regulatory Modules for Lung Adenocarcinoma

#### 2.3.1. Candidate mRNA Selection

In order to select candidate mRNAs that not only contributed significantly to the mRNA DNN models, but also were in the center of the interaction network, their contribution to mRNA DNN models and centrality values were jointly analyzed by Jointplot of the python Seaborn module. Jointplot is a Seaborn function that plots a scatter graph for two variables with distinct histograms at the plot’s upper edge and right sides.

On the one hand, the contribution of each mRNA to the mRNA DNN models was evaluated by SHAP values. On the other hand, central mRNAs were identified by the plug-in cytoHubba of Cytoscape using Maximal Clique Centrality (MCC) algorithm since MCC had a better performance in predicting PPI network central nodes among all the centrality measures [21]. Therefore, Candidate mRNAs were selected as those with high values of both measures.

#### 2.3.2. Potential ceRNA Screening

In order to reveal the ceRNA regulatory mechanism in lung adenocarcinoma, a ceRNA DNN model was constructed. SHAP values were also used to evaluate the contribution of each RNA (mRNA, miRNA or lncRNA) to the ceRNA DNN model. RNAs with SHAP value > 0.0001 were screened out as potential ceRNAs and used to reconstruct a potential ceRNA subnetwork with ceRNA regulatory and mRNA interaction relationships.

#### 2.3.3. Regulatory Module Identification and Validation

Modules comprising one miRNA and its regulated lncRNAs and mRNAs with the same up/down regulation direction were selected from the potential ceRNA subnetwork as regulatory modules for lung adenocarcinoma.

Literature review was conducted by searching in the PubMed database for all articles published in English Language on the topics of the identified regulatory modules and lung adenocarcinoma.

The metascape platform was used to conduct functional enrichment analysis based on GO, KEGG, wikiPathways and Hallmark databases for mRNAs in regulatory modules for lung adenocarcinoma. Categories with the minimum overlap number 3 and the hypergeometric test Benjamini–Hochberg adjusted *p*-value < 0.05 were selected.

In order to reflect the effectiveness of identified regulatory modules for lung adenocarcinoma, 551 unpaired samples from the lung adenocarcinoma dataset from The Cancer Genome Atlas (TCGA-LUAD) (including 497 tumor and 54 paracancerous non-tumor samples, completely different from the training dataset) were used as an independent dataset. Traditional machine learning methods, including K-nearest neighbor (KNN), Support Vector Machine (SVM), decision tree, Multi-feature Bayesian, Logistic regression, and random forest were also applied to sample classification using identified regulatory modules. Their performance was compared to that of the DNN model by area under receiver operating characteristic curves (AUC) values of the area under the receiver operating characteristic (ROC) curves.

## 3. Results

### 3.1. Candidate mRNAs

**Figure 2:**
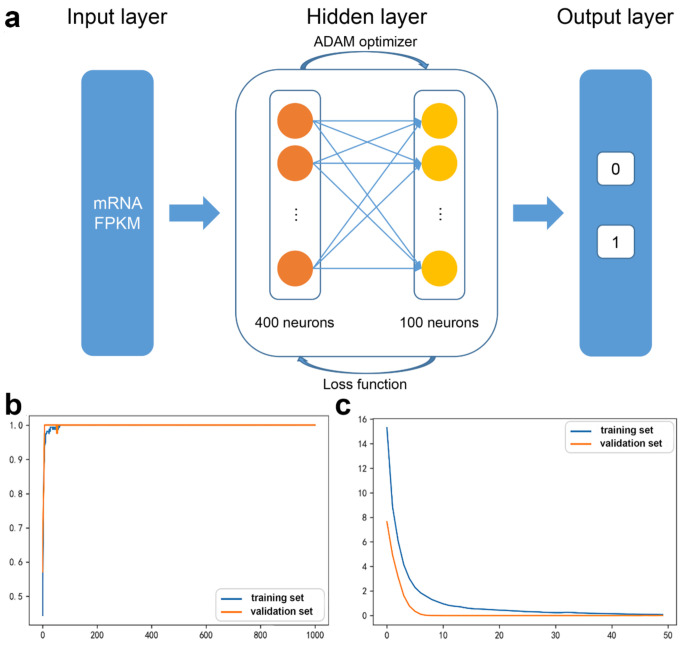
The mRNA DNN model: (**a**) The DNN model structure, (**b**) accuracy curve, and (**c**) loss curve.

For the mRNA DNN models, the initial input layer was set to the FPKM values of 1122 mRNAs from the interaction network, and two hidden layers of 400 and 100 neurons were established, with the 0/1 label as the output layer (Figure 2a).

The accuracy curve and the loss curve were both accordant with the general law of deep learning (Figure 2b,c). The loss curve for the training set and the validation set decreased with the increase in iteration number. However, when the training times were between 5 and 10 the loss for the training set decreased, while for the validation set it was stable, indicating the occurrence of overfitting.

**Figure 3:**
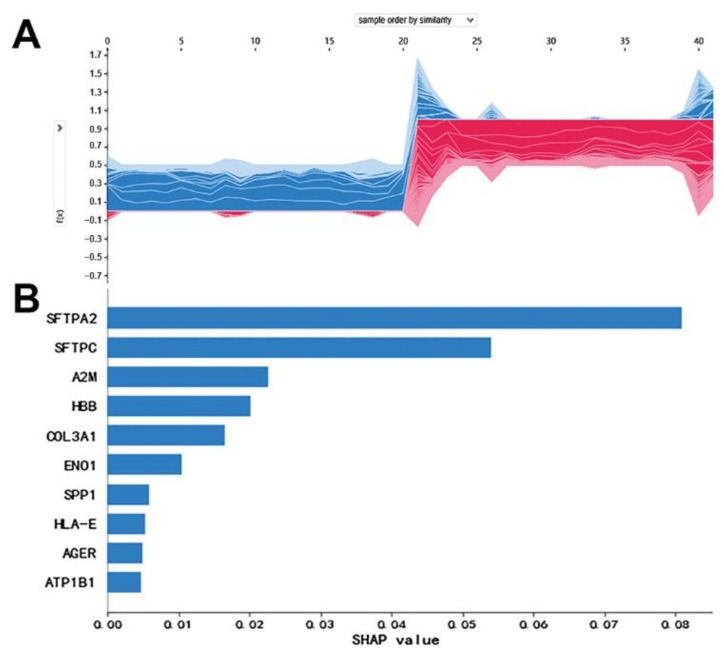
The SHAP values of the mRNAs: (**A**) Contribution of each mRNA to partial individual sample. Red represents the positive influence, and blue represents the negative influence. The abscissa represents the samples, and the ordinate represents the SHAP values. (**B**) The SHAP values of top 10 mRNAs based on their contribution to all samples.

In order to alleviate the imbalance of each feature and eliminate the phenomenon of overfitting, regularization layers were added after each hidden layer. The python SHAP module was applied to interpret the contribution of each mRNA to the DNN model (Figure 3).

**Figure 4:**
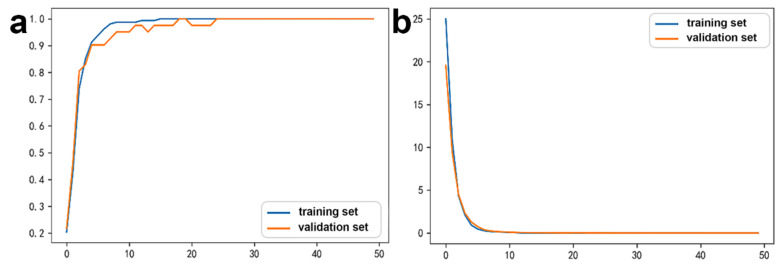
The accuracy and loss of the optimized mRNA DNN model after removing mRNAs with SHAP value = 0. (**a**) Accuracy curve and (**b**) loss curve.

The 18 mRNAs with SHAP value = 0 were removed and the DNN model was relearned (Figure 4). The accuracy was 97.04%. The validation loss was slightly greater than the training loss, and the two curves tended to converge. Hence, the regularization optimization was effective.

**Figure 5:**
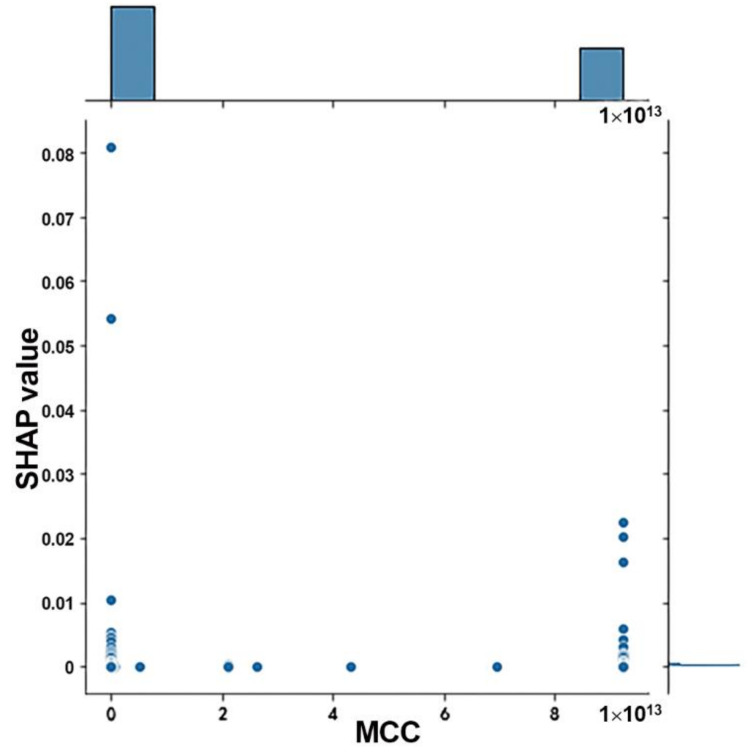
The joint distribution of SHAP and MCC values. MRNAs are mainly clustered in two categories.

In order to select candidate mRNAs that had not only high contribution on DNN models, but also central properties in the interaction network, the SHAP values of DNN models and the MCC values of network nodes were jointly analyzed. It was demonstrated that the mRNAs were mainly clustered in two categories (Figure 5). Therefore, a total of 699 mRNAs with MCC > 8 and SHAP value > 10^−4^ were selected as candidate mRNAs.

### 3.2. Potential ceRNAs

Based on miRNA target information, candidate mRNAs obtained by previous DNN models and significant differentially expressed lncRNAs targeted by significant differentially expressed miRNAs were extracted. Pearson correlation coefficients of miRNA-lncRNA, miRNA-mRNA and mRNA-lncRNA were calculated to screen ceRNA triplets (*p*-value < 0.05). Finally, 518 ceRNAs triplets were screened out, containing 309 mRNAs, 13 miRNAs and 12 lncRNAs. Combing the interaction network and screened ceRNA triplets, a ceRNA network comprised of 270 mRNAs, 13 miRNAs and 12 lncRNAs was constructed after removing isolated nodes.

**Figure 6:**
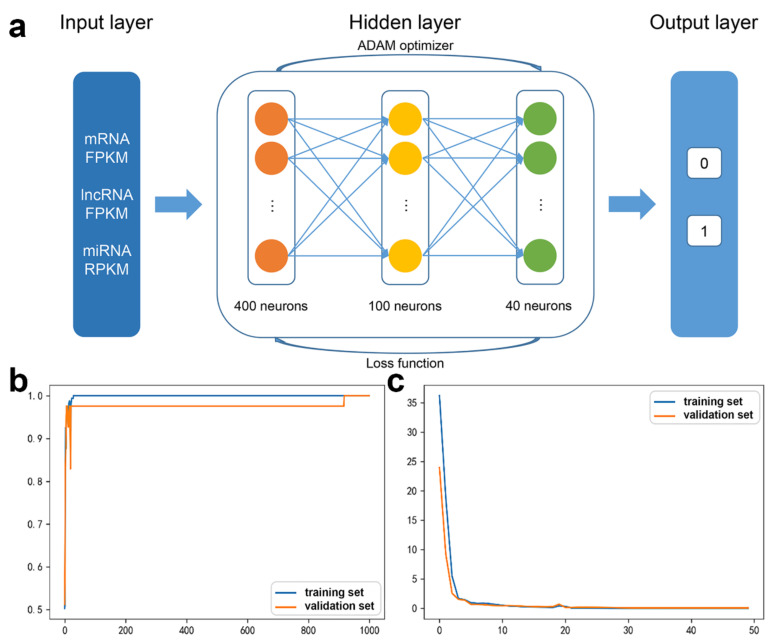
The ceRNA DNN model: (**a**) The DNN model structure, (**b**) accuracy curve, and (**c**) loss curve.

To achieve a better performance, the regularization layers in the previous optimized mRNA DNN model were removed and a layer of 40 neurons was added to expand the capacity of the neural network. FPKM values for 13 miRNAs and 12 lncRNAs were used as features for the ceRNA DNN model (Figure 6a). The accuracy curve finally converged, and the loss curve uniformly converged to 0 (Figure 6b,c). Therefore, the ceRNA DNN model was feasible and appropriate.

**Figure 7:**
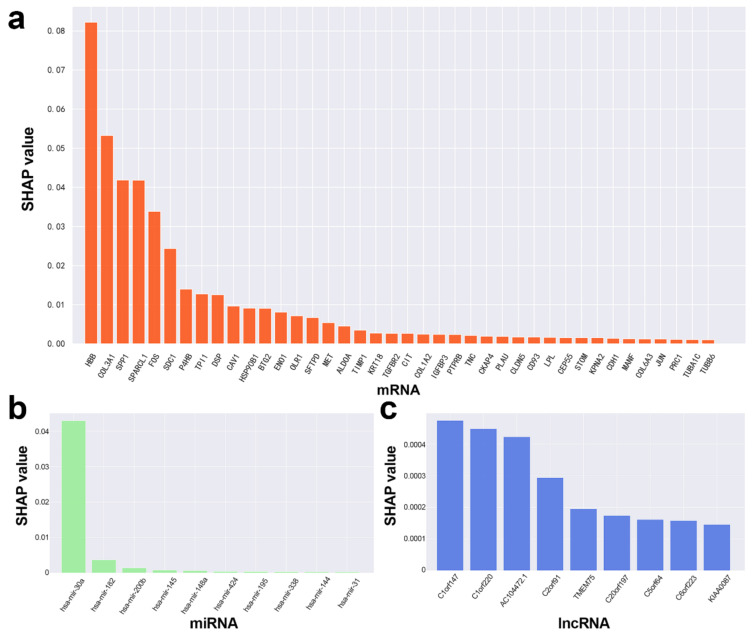
The SHAP values of top RNAs with SHAP value > 0.0001 in the ceRNA DNN model: (**a**) Top 40 mRNAs, (**b**) top 10 miRNA, and (**c**) top 9 lncRNA.

**Figure 8:**
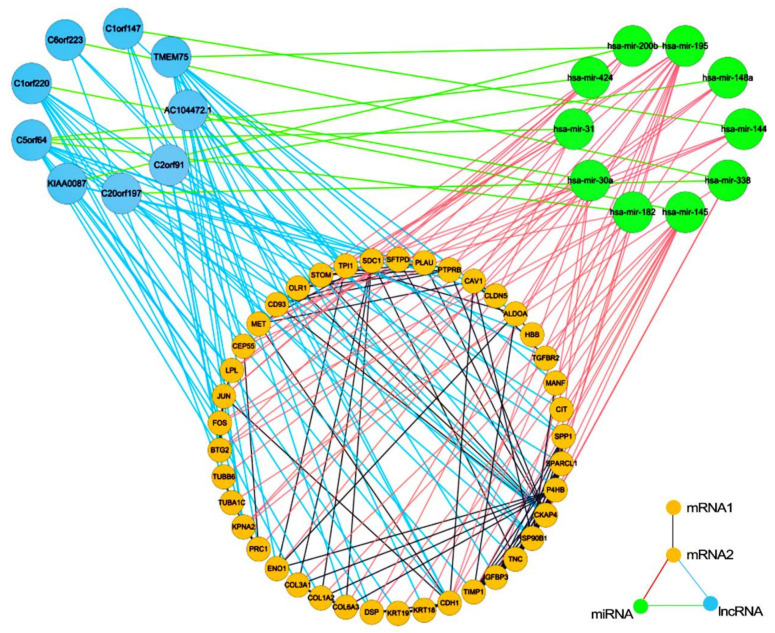
The potential ceRNA subnetwork with ceRNA regulatory and mRNA interaction relationships.

SHAP values were used to evaluate the contribution of each RNA (mRNA, miRNA or lncRNA) to the ceRNA DNN model. RNAs with SHAP value > 0.0001 (including 40 mRNAs, 10 miRNAs and 9 lncRNAs) were screened out as potential ceRNAs (Figure 7) and used to reconstruct the potential ceRNA subnetwork with ceRNA regulatory and mRNA interaction relationships (Figure 8).

**Figure 9:**
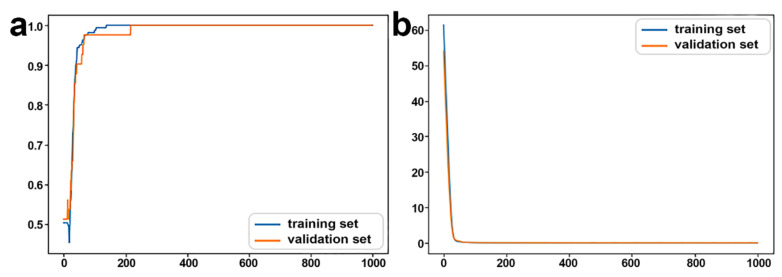
The accuracy and loss of the ceRNA DNN model for potential ceRNAs: (**a**) Accuracy curve and (**b**) loss curve.

With FPKM of these potential ceRNAs as features for the input layer, another ceRNA DNN model was established and trained. The accuracy increased and the loss decreased as the training progressed (Figure 9), indicating no overfitting during the process. The learning process conformed to the law of deep learning, and the learning efficiency of deep learning ensured the reliability of these potential ceRNAs.

In the DisGeNet database (https://disgenet.org/, accessed on 9 April 2021), 37 potential mRNAs/genes were cancer/tumor related genes, among which 25 were directly related to lung tumors and complications. GO functional enrichment analysis showed that potential mRNAs mainly enriched in GO-BP functions such as regulated exocytosis, transforming growth factor beta receptor signaling, muscle structure development, and response to extracellular stimulus, referring to the phenomenon of metastases and lung tissue deterioration during cancer development. The functions of GO-MF were mainly cell adhesion molecule binding, SMAD binding and other membrane binding protein activity. These potential mRNAs were mainly enriched in functions directly related to the proliferation, metastasis and diffusion of cancer cells, and were highly correlated with the occurrence and development of lung adenocarcinoma.

### 3.3. Regulatory Modules for Lung Adenocarcinoma

**Figure 10:**
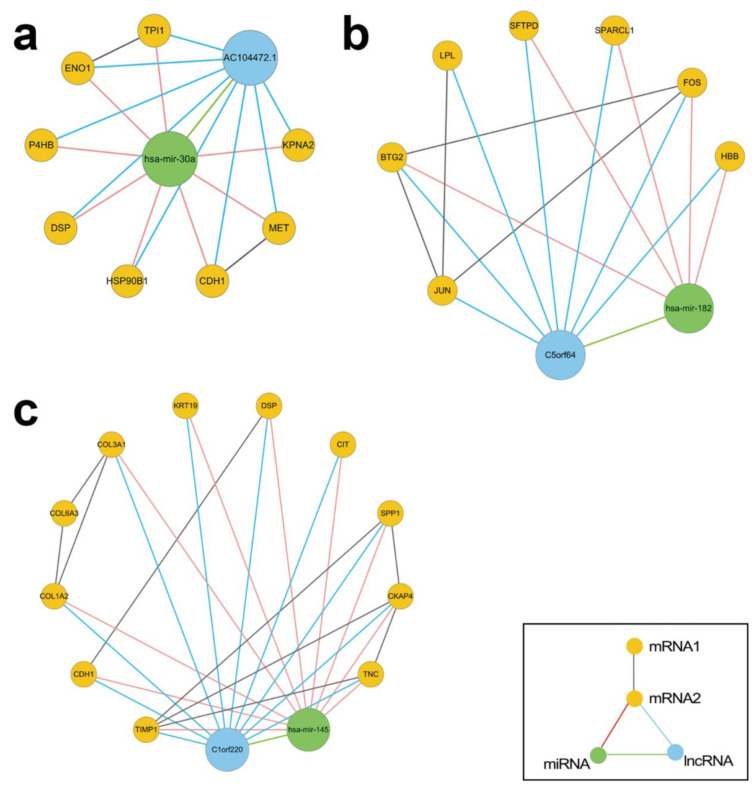
Three regulatory modules for lung adenocarcinoma. (**a**) regulatory module for lung adenocarcinoma a: hsa-mir-30a with its regulated lncRNA AC104472.1 and mRNAs, (**b**) regulatory module for lung adenocarcinoma b: hsa-mir-182 with its regulated lncRNA C5orf64 and mRNAs, and (**c**) regulatory module for lung adenocarcinoma c: hsa-mir-145 with its regulated lncRNA C1orf220 and mRNAs.

Modules comprised of one miRNA and its regulated lncRNAs and mRNAs with the same up/down regulation direction were identified as regulatory modules for lung adenocarcinoma (Figure 10). MiRNA hsa-mir-30a and lncRNA AC104472.1 constituted regulatory module for lung adenocarcinoma a with TPI1, KPNA2, MET, HSP90B1, P4HB, DSP, CDH1, and ENO1 (Figure 10a). Among them, hsa-mir-30a had the highest contribution in potential miRNAs, and its expression level was significantly down-regulated. Hsa-mir-182, which ranked second in potential miRNA contribution, and lncRNA C5orf64 formed regulatory module for lung adenocarcinoma b (Figure 10b). Among the RNAs regulated by hsa-mir-182, the hemoglobin β coding gene HBB has the highest contribution in potential mRNAs. Hsa-mir-145 formed the third regulatory module for lung adenocarcinoma c with lncRNA C1orf220, which ranked second in potential lncRNAs, and mRNAs (COL1A2, COL3A1, SPP1, TIMP1 and CDH1) (Figure 10c).

These regulatory modules for lung adenocarcinoma were further validated by literature review, functional enrichment analysis and an independent dataset.

#### 3.3.1. Literature Review

Related studies have shown that hsa-mir-30a, as an important regulator of tumor suppressors, could inhibit cell proliferation, migration, and invasion in vitro [22]. When its expression is down-regulated, cancer is more likely to become worse. The lncRNA AC104472.1 is one of the cancer lncRNAs of the immune-related function, and is a potential prognostic marker for the treatment of breast cancer [23]. Additionally, hsa-mir-30a has a potential role in regulating autophagy in cancer cells. Autophagy-related genes (e.g., P4HB) are overexpressed if hsa-mir-30a regulation is inhibited. Accelerated autophagy behavior of normal cells provides a microenvironment enriched with nutrients for cancer cells [24]. Current studies have shown that P4HB is overexpressed in all kinds of tumor cells and is an important indicator to detect the tumor progression level [25]. In addition to P4HB, the mRNA targets regulated by hsa-mir-30a also included many cancer therapeutic targets. For example, DSP is related to the growth and metastasis of cancer cells [26]. CDH1 is a widely known proto oncogene in the occurrence and development of cancer [27], while a relatively new oncogenic determinant MET regulates the occurrence, progression, and malignancy of epithelial carcinomas including lung adenocarcinoma [28].

Hsa-mir-182 was upregulated in lung and other cancers to promote cancer cell migration and invasion, and was found to have good potential for cancer diagnosis [29]. lncRNA C5orf64, which was regulated by hsa-mir-182, has recently been confirmed by a large number of bioinformatics methods to be significantly positively correlated with the abundance of immune neutrophils, and has the potential to regulate tumor microenvironment and help to reshape mutant patterns [30]. In lung adenocarcinoma, the dysfunction of HBB can directly lead to different degrees of anemia in patients, which has become a major problem in the treatment of lung adenocarcinoma [31]. In addition, BTG2 is a gene enriched in the Hallmark of angiogenesis and platelets, whose downregulation is directly related to the invasion of cancer cells [32]. SPARCL1, which was also down-regulated by hsa-mir-182, was proved to be able to optimize clinical efficacy by preventing tumor invasion and angiogenesis [33].

LncRNA C1orf220 was targeted by hsa-mir-145. Bioinformatics studies have shown that C1orf220 plays an important role in central gene regulation of lung squamous cell carcinoma [34]. COL3A1 and COL1A2 higher expressed in tumor samples were hub genes in a miRNA–gene interaction network and related to the survival time of lung adenocarcinoma [35]. Silencing SPP1 was found to reduce EGFR resistance to tyrosine kinase inhibitors and reduce its invasiveness in lung adenocarcinoma [36]. Tumor-derived protein tissue inhibitor of metalloproteinases-1 (TIMP1) correlates with poor prognosis in many cancers [37]. Wang et al. confirmed that TIMP1 regulated metabolism in metastases by activating the PI3K/Akt pathway and found TIMP1 as a potential biomarker for understanding lung adenocarcinoma pathogenesis [38].

These regulatory modules for lung adenocarcinoma provided a better insight into the regulatory role they play in the initiation of lung adenocarcinoma.

#### 3.3.2. Functional Enrichment Analysis

**Figure 11:**
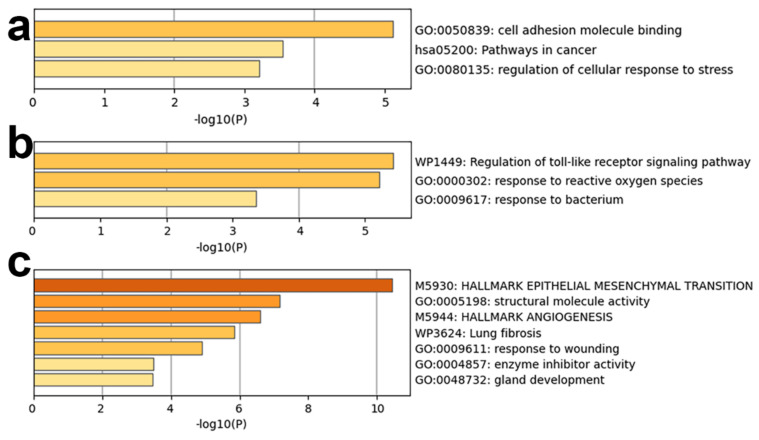
Functional enrichment analysis results of mRNAs in three regulatory modules for lung adenocarcinoma: (**a**–**c**) are results for three regulatory modules for lung adenocarcinoma a, b, and c, respectively.

GO, KEGG, wikiPathways and Hallmark functional enrichment analysis was performed for mRNAs in regulatory modules for lung adenocarcinoma (Figure 11). GO functional enrichment analysis showed that mRNAs in each module mainly enriched in GO functions such as cell adhesion molecule binding, response to reactive oxygen species, enzyme inhibitor activity, etc. These functions are all critical factors involved in cancers. The regulation roles of them in lung adenocarcinoma has been studied [39,40,41].

For KEGG pathway enrichment analysis, mRNAs in the first regulatory module for lung adenocarcinoma were mainly enriched in pathways in cancer. In addition, the results of enrichment by wikiPathways showed that mRNAs in the other two regulatory modules for lung adenocarcinoma were mainly enriched in Regulation of toll-like receptor signaling pathway and Lung fibrosis. Toll-like receptors (TLRs), such as Toll-Like Receptor 2 and 4, are pillars of the immune system that have been linked to several forms of malignancy including lung adenocarcinoma [42,43]. Lung fibrosis has been reported to be a risk factor for developing lung carcinogenesis [44].

MRNAs in the third regulatory module for lung adenocarcinoma were enriched mainly in the Hallmark gene set of EPITHELIAL MESENCHYMAL TRANSITION and ANGIOGENESIS. Lung adenocarcinoma could cause severe epithelial-mesenchymal transition disorder, leading to pulmonary epithelial cell dysfunction [45]. Angiogenesis is the process of capillary sprouting from pre-existing vessels and plays a critical role in the carcinogenic process of lung adenocarcinoma [46].

#### 3.3.3. Independent Dataset Validation

**Figure 12:**
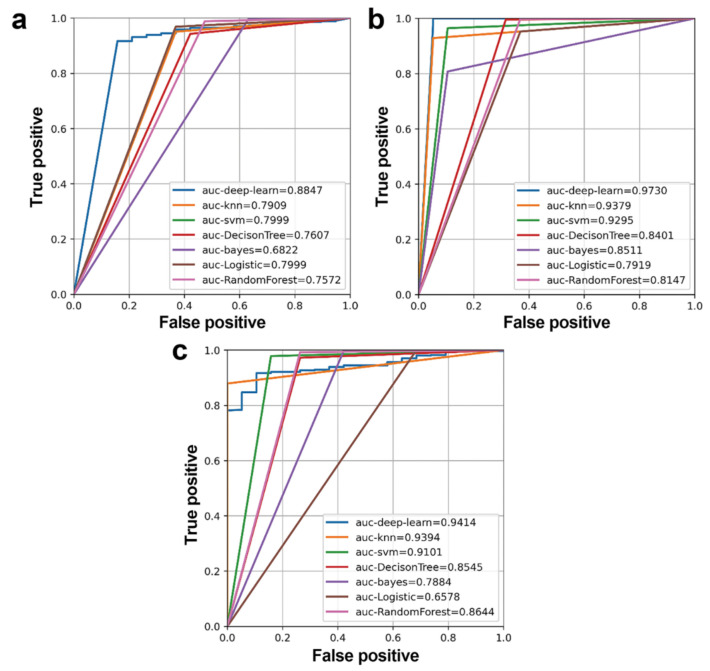
ROC curves and AUC values of regulatory modules for lung adenocarcinoma for the independent dataset using different machine learning methods. (**a**–**c**) are results for three regulatory modules for lung adenocarcinoma a, b, and c, respectively.

To further demonstrate the effectiveness of identified regulatory modules for lung adenocarcinoma, the DNN model was applied to classify samples of an independent dataset TCGA-LUAD using three regulatory modules for lung adenocarcinoma, respectively. In order to further prove that the DNN model was superior to traditional machine learning methods, KNN, SVM, decision tree, Multi-feature Bayesian, Logstic regression, and random forest were applied to the independent dataset, respectively. The ROC curve was drawn according to a series of different cut-off values with true positive as the ordinate and false positive as the abscissa (Figure 12).

Most machine learning methods had good classification performance (AUC > 0.75), while the DNN model had the best performance, demonstrating the effectiveness and diagnostic values of all three regulatory modules for unpaired lung adenocarcinoma samples.

## 4. Discussion

In this paper, three regulatory modules for lung adenocarcinoma were identified from multiple DNN models using expression data in interaction and ceRNA networks. They participated in the carcinogenesis of lung adenocarcinoma by regulation of miRNAs to mRNAs and lncRNAs. These modules were further validated in literature and an independent dataset and were expected to be used for lung adenocarcinoma diagnosis. The main advantage of DNN is that it can modify the multidimensional weight of each feature during the learning process. Regulatory modules for lung adenocarcinoma identified using DNN models from paired samples of CPTAC could distinguish disease samples from normal ones for unpaired samples of the TCGA-LUAD dataset, indicating the effectiveness for both paired and unpaired samples. The higher accuracy might come from the power of DNN in describing sophisticated relationships between genes, while the simple classification rules that traditional machine learning methods used may be not capable enough. The novelty of our study was summarized as follows: (1) The expression data was acquired from CPTAC and TCGA, which were all large multi-omic datasets for various cancers. This made our results covered most scenario.; (2) The ceRNA network constructed based on experimentally validated miRNA targets and correlations could better explain the respective roles of different RNAs in biological processes; (3) Potential RNAs were screened by multiple mRNAs and ceRNAs DNN models, which could intelligently recognize the sophisticated relationships between RNAs appropriately.

Regulatory modules for lung adenocarcinoma regulated the initiation process of lung adenocarcinoma through ceRNAs relationships. All mRNAs of regulatory modules for lung adenocarcinoma have been validated to be associated with cancer in DisGeNet, of which 15 were directly related to lung cancer and its complications. LncRNAs and miRNAs were further searched in ncRNA-disease association databases to further exhibit their disease association. Two lncRNAs of regulatory modules for lung adenocarcinoma were stored in LncRNADisease v2.0 as non-small cell lung cancer associated, which integrated comprehensive experimentally supported and predicted ncRNA-disease associations curated from manual literatures and other resources [47]. All miRNAs of regulatory modules for lung adenocarcinoma were associated with lung adenocarcinoma in HMDD (the Human microRNA Disease Database) v3.2 curating experimentally supported miRNA and disease association data [48]. Experimental validation is needed for other RNAs in regulatory modules for lung adenocarcinoma in the future.

The regulatory modules for lung adenocarcinoma we identified were not population specific, since the CPTAC data contained 66 white, 2 black or African American, 2 Asian, and 130 not reported samples according to the race data from the clinical information. To study the regulatory relationships of RNAs in the three regulatory modules for specific population, we recalculated the correlations between RNAs for different races respectively. Most RNA pairs for white samples showed the same regulatory relationships (the black or African American and Asian samples were omitted because the number of them were too small to be correlated, Table S1). Then similar progress was performed to the TCGA-LUAD dataset according to the race data, i.e., 456 white, 58 black or African American, 8 Asian and 29 not reported samples. Similar results were obtained for most cases of white and black or African American samples (Table S1). Regulatory relationships for Asian samples (the correlation analysis was not performed for hsa-mir-30a since its expression value in Asian samples is zero) were not the same as those in the regulatory modules, probably due to the small sample size. As a result, we searched for other datasets with miRNA, mRNA and lncRNA expression data of Asian samples in the GEO database, but none was obtained. Only one expression profile with mRNA and miRNA expression data, GSE128311, was found. This dataset had 77 Asian samples. It was found that the regulatory relationships of GSE128311 were consistent with the regulatory relationships in the three regulatory modules (only the correlations between miRNAs and mRNAs were calculated, as no lncRNA was in this dataset, Table S1). The above results did not illustrate the population specificity of the regulatory modules for lung adenocarcinoma.

**Figure 13:**
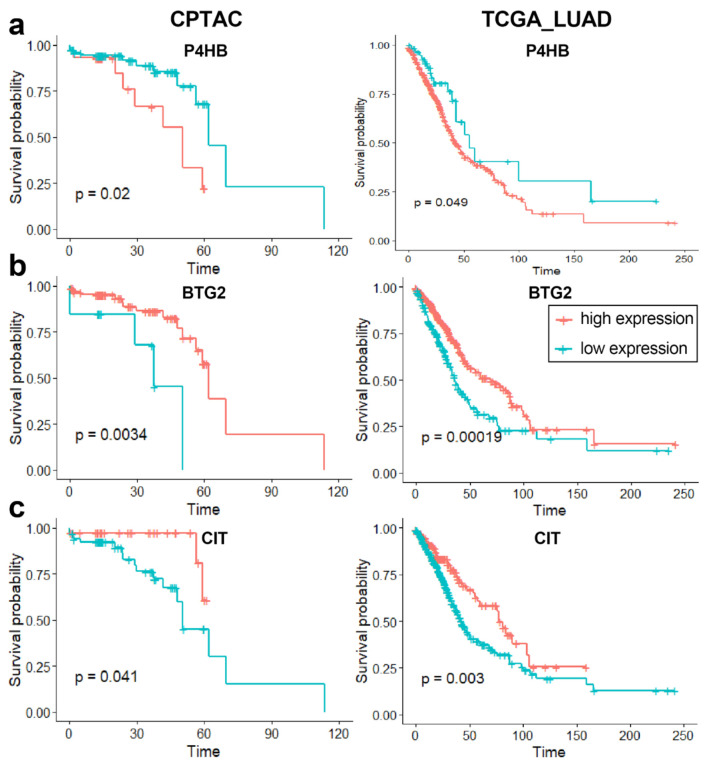
Kaplan–Meier survival analysis results for (**a**) P4HB, (**b**) BTG2 and (**c**) CIT in CPTAC and TCGA-LUAD datasets.

The prognosis value of regulatory modules for lung adenocarcinoma was then evaluated using the univariate Kaplan–Meier survival analysis and multivariate Cox regression. Kaplan–Meier curve showed similar results for some mRNAs of regulatory modules for lung adenocarcinoma in CPTAC and TCGA-LUAD datasets. For example, high expression of P4HB as well as low expression of BTG2 and CIT was associated with poor overall survival of lung adenocarcinoma patients (*p*-value of log-rank test < 0.05, Figure 13). Clinical application of these genes was worth further study due to their diagnostic and prognostic value.

Other RNAs of regulatory modules for lung adenocarcinoma showed better results in the TCGA-LUAD dataset. Kaplan–Meier survival analysis in the TCGA-LUAD dataset showed that the overall survival of patients with the low expression hsa-mir-30a was poorer than that with the high expression (*p* < 0.05). Moreover, multivariate Cox analysis confirmed that each module was a risk factor for overall survival among patients in the TCGA-LUAD cohort (*p* < 0.05). A probable reason for better TCGA-LUAD prognostic results was that it was a more balanced dataset for different stages, while most patients of CPTAC were in their early stage. In spite of this, regulatory modules for lung adenocarcinoma could still be used for survival analysis in more balanced datasets for different stages. Further experimental validation for the regulatory relationships in lung adenocarcinoma would also be necessary.

In this paper, ceRNA networks were constructed according to experimentally validated miRNA targets from multiple databases, making the size of ceRNA networks small. The interactions predicted by confident computational tools should be taken into the analysis to improve the effectiveness and reliability of regulatory modules for lung adenocarcinoma.

## 5. Conclusions

To sum up, three regulatory modules for lung adenocarcinoma were identified using expression data by multiple DNN models in biological networks. MiRNAs hsa-mir-30a, hsa-mir-182, hsa-mir-145 and their regulated lncRNAs and mRNAs participated in the carcinogenesis of lung adenocarcinoma through ceRNAs relationships. These regulatory modules showed the relationship with lung adenocarcinoma in terms of expression levels, functions, pathways, and literature. They could distinguish disease samples from normal ones, and thus had potential for lung adenocarcinoma diagnosis. The regulatory relationships between RNAs were validated in various datasets, including CPTAC, TCGA and an expression profile from the GEO database. These regulatory modules had potential values for clinic prognosis. Our study will contribute to improving the understanding of the ceRNA network regulatory mechanisms in the carcinogenesis of lung adenocarcinoma and provide schemes for identifying novel regulatory modules of other cancers.

## Figures and Tables

**Table 1:** The pathological characteristics of the lung adenocarcinoma patients.

	CPTAC
**Patient (n)**	100
**Age, years**	
median	63.5
range	35–81
**Sex (%)**	
male	63 (63%)
female	37 (37%)
**Tumor_grade (%)**	
G1	7 (7%)
G2	55 (55%)
G3	37 (37%)
GX	1 (1%)
**Ajcc_pathologic_stage (%)**	
Stage I	54 (54%)
Stage II	29 (29%)
Stage III	17 (17%)

## Data Availability

Publicly available datasets were analyzed in this study. This data can be found here: https://proteomics.cancer.gov/programs/cptac (accessed on 20 March 2021).

## Supplementary Materials

The following supporting information can be downloaded at: https://www.mdpi.com/article/10.3390/biology11091291/s1, Table S1: The regulatory relationships of the regulatory modules for different races in CTPAC, TCGA and GSE128311.
